# Influence of Warm-Up Music Preference on Anaerobic Exercise Performance in Division I NCAA Female Athletes

**DOI:** 10.3390/jfmk6030064

**Published:** 2021-07-23

**Authors:** Corinne E. Meglic, Caroline M. Orman, Rebecca R. Rogers, Tyler D. Williams, Christopher G. Ballmann

**Affiliations:** Department of Kinesiology, Samford University, Birmingham, AL 35226, USA; cmeglic@samford.edu (C.E.M.); corman@samford.edu (C.M.O.); rrogers1@samford.edu (R.R.R.); twilli11@samford.edu (T.D.W.)

**Keywords:** Wingate, preferred music, motivation, power output, rate of perceived exertion

## Abstract

The purpose of this study was to investigate the effects of listening to preferred versus non-preferred warm-up music on anaerobic sprint performance in Division I NCAA female athletes. Female collegiate athletes (*n* = 14) were recruited for this study. In a counterbalanced, crossover study design, participants completed two separate visits, each with a different warm-up music condition: preferred (Pref) or non-preferred (Non-pref). During each visit, participants completed a 3 min standardized cycling warm-up at 50 Watts while listening to Pref or Non-pref music. Following this, participants completed 3 × 15 s Wingate Anaerobic Tests (WAnTs) with a 2 min active recovery period in between tests. Motivation to exercise was measured immediately following the warm-up (WU), WAnT1, WAnT2, and WAnT3. The rate of perceived exertion (RPE) was also measured after each WAnT. Each visit was separated by a minimal recovery period of 48 h. Mean power, total work, RPE, and motivation were analyzed. Mean power (*p* = 0.044; d = 0.91) and total work (*p* = 0.045; d = 0.78) were significantly higher during the Pref music condition versus Non-pref. RPE remained unchanged regardless of condition (*p* = 0.536; d = 0.01). Motivation was significantly higher with Pref warm-up music compared to Non-pref (*p* < 0.001; d = 1.55). These results show that listening to Pref warm-up music has an ergogenic benefit during repeated sprints in comparison to Non-pref music and improves motivation to exercise. Listening to warm-up music prior to high-intensity repeated exercise may aid in optimizing performance and training in collegiate athletes.

## 1. Introduction

Music has been widely studied for its ergogenic effects across multiple modes of exercise [1,2]. Through psychological, physiological, and psychophysiological mechanisms, music has been shown to impart increases in muscular endurance, strength, and cardiorespiratory exercise performance [2]. Various factors may determine the efficacy of music on performance, including intrinsic characteristics of songs (i.e., tempo, volume), subjective preference (i.e., preferred or non-preferred), and timing of music application (i.e., before or during exercise) [1]. How these factors affect one another, resulting in performance outcomes, remains to be fully elucidated, especially in the context of anaerobic exercise.

Multiple studies have indicated that listening to warm-up music significantly improves anaerobic exercise performance, although some literature is conflicting [3,4,5,6]. Chtourou et al. reported enhanced power output in male sprinters during a 30 s Wingate Anaerobic Test (WAnT) following a 10 min warm-up with music versus no music [4]. Supporting this, Jarraya et al. showed increases in the peak and mean power levels during a 30 s WAnT after listening to warm-up music in well-trained athletes [6]. However, others have shown little or no improvement in anaerobic performance with warm-up music [5,7]. Fox et al. showed no changes in power output during a 30 s WAnT after listening to warm-up music in both males and females [5]. Reasons for disparities between findings are not fully clear but may be due to differing music selection protocols and subjective preference of music by participants. 

Music preference unequivocally influences the efficacy of the ergogenic potential of music [1,8,9,10,11,12,13]. Nakamura et al. reported increases in endurance cycling performance and dissociation while listening to preferred (Pref) music compared to non-preferred (Non-pref) [14]. Our lab has shown increases in repetition volume and barbell velocity during bench pressing while listening to Pref music [12]. Furthermore, we have also shown listening to Pref music increases motivation and dissociation during repeated sprints [11]. Indeed, many of these effects are likely mediated by increases in exercise motivation with Pref versus Non-pref music [8,10,12,13]. For a comprehensive examination of music preference and exercise, the reader is directed to a recent review by Ballmann [1]. From this, it is clear that music preference and anaerobic exercise performance are understudied, thereby necessitating the need for further explication of how Pref or Non-pref music may modify anaerobic performance.

To date, only two studies have investigated the effects of warm-up music preference on exercise performance [8,13]. Recently, our group showed that listening to Pref warm-up music improved endurance rowing performance and increased motivation to exercise, versus Non-pref music, in physically active males and females [13]. We also observed increases in bench press repetition volume, and motivation to exercise when listening to Pref versus Non-pref music in resistance-trained males [8]. However, it is unknown if preferred warm-up music influences repeated sprint ability and whether possible effects are manifested in psychological or psychophysiological mechanisms. Thus, the purpose of this study was to investigate the effects of listening to Pref versus Non-pref warm-up music on anaerobic sprint ability, motivation to exercise, and RPE in collegiate female athletes.

## 2. Materials and Methods

### 2.1. Participants

To determine the appropriate sample size, an a priori power analysis was conducted using G-power 3.1.9.6 software. A previous investigation from our lab showed improvements in repetitions during repeated bench press exercises following listening to Pref warm-up music with an effect size of *f* = 0.902 [8]. Therefore, adequate sample size was calculated using the following parameters: test-repeated measures ANOVA, *f* = 0.902, α = 0.05, β = 0.8, groups = 2, measurements = 3, correlation = 0.5. This equated to a minimum sample size of *n* = 6. To be comparable in sample size to other studies [8,11,12], fourteen female Division I National Collegiate Athletic Association (NCAA) collegiate soccer (*n* = 7) and volleyball (*n* = 7) athletes volunteered to participate, and their descriptive characteristics are shown in Table 1. To be considered an NCAA athlete, all participants had to be on an active Division I roster in the past year [9,15]. A physical activity readiness questionnaire (PAR-Q) was taken prior to completing the study, ensuring safety for exercise [16]. All participants were free of a lower-body injury within the past six months before participation. Participants were asked not to consume alcohol, nicotine, or caffeine 12 h prior and refrain from vigorous physical activity 24 h prior to completing the study.

### 2.2. Preferred (Pref) and Non-Preferred (Non-Pref) Music Determination

During each participants’ first visit, they completed a single survey on music preference as previously described by our lab [1,10,13]. Briefly, five different genres, including rap/hip hop, country, pop, dance electronic, and rock and roll, were rated from most preferred to least preferred. For the Pref music condition, participants self-selected a song from their most preferred genre as long as it had a tempo of ≥120 bpm. For the Non-pref music condition, a tempo-matched song was chosen by the researchers from the participants’ least favorite genre. Music was listened to through headphones at the same volume level for all participants [8]. 

### 2.3. Protocol

Participants completed 2 visits, each with a different warm-up music condition: (1) Pref, (2) Non-pref. Participants completed a 3 min cycling warm-up on a cycle ergometer (Monark, Healthcare International, Langley, WA, USA) at 50 Watts while listening to the corresponding music condition. Following this, the music was stopped, and participants performed 3 × 15 s repeated Wingate Anaerobic Tests (WAnT) on an electronically braked cycle ergometer (Velotron, Racermate Inc., Seattle, WA, USA) [11,17]. The seat height was modified according to the participant’s height and then was recorded for repeatability for the following visit. Pedaling resistance was set at 7.5% of the participant’s body mass. Once the 3 min warm-up was done, the music was stopped. Each WAnT began with a 10 s lead-in phase to allow participants to achieve a maximal pedaling rate. Following this, resistance was immediately added, and the participant pedaled for 15 s maximally. Each WAnT was separated by a 2 min active recovery period in which participants pedaled, self-paced, at an unloaded resistance. WAnT procedures were repeated for a total of 3 tests. Following each WAnT, both the rate of perceived exertion (RPE) and motivation to exercise were measured. RPE was measured on a 1–10 scale, where 1 indicated “extremely easy” and 10 indicated “so hard cannot continue”. The motivation was recorded using a visual analog scale with a 100 mm line. The participant rated their motivation on a scale from 0 to 100 mm, with 0 being “no motivation” and 100 being “extremely motivated” [8,10,11,12]. Performance variables were calculated from each trial using Velotron software (v4 1.0.6 Velotron, Racermate Inc., Seattle, WA, USA). 

### 2.4. Data Analysis

All data were analyzed using Jamovi software (Version 0.9; Sydney, Australia). Test-to-test differences for all variables were detected using a 2 × 3 [condition × test] repeated measures ANOVA with a Tukey post-hoc. Average performance (AVG) over the 3 WAnTs, which represents the main effects for condition, are also shown in figures. Estimates of effect size for main effects were calculated using partial eta squared (η^2^_p_) and interpreted as: 0.02—small; 0.13—medium; ≥0.26—large [18,19]. For mean differences, effect sizes were calculated via Cohen’s d (d) between and interpreted as: 0.2—small; 0.5—moderate; 0.8—large [18,19]. All data are presented as mean ± standard deviation (SD). Significance was set at *p* ≤ 0.05 a priori. 

## 3. Results

### 3.1. Anaerobic Performance

**Figure 1:**
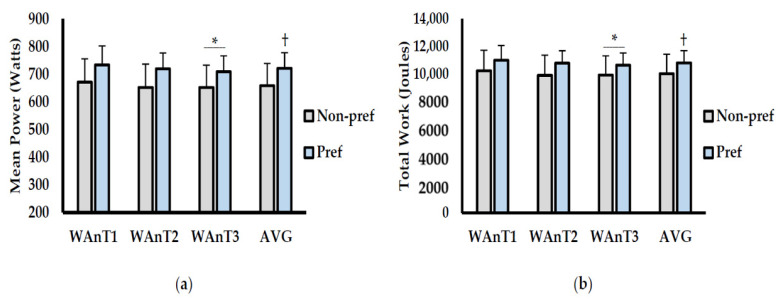
(**a**) Mean power (watts) and (**b**) total work (joules) between non-preferred (Non-Pref; grey bars) and preferred (Pref; blue bars) warm-up music conditions. Measurements are shown for WAnT1, WAnT2, WAnT3, and the average of all three tests together (AVG) for each condition. Data are presented as mean ± SD. * indicates significantly different from WAnT1 (*p* ≤ 0.05). † indicates significantly different than Non-pref (*p* ≤ 0.05).

Anaerobic performance outcomes are displayed in (Figure 1). For mean power (Watts; Figure 1a), there was a main effect for condition (*p* = 0.044; η^2^_p_ = 0.27) and test (*p* = 0.016; η^2^_p_ = 0.27). No interaction between condition × test (*p* = 0.468; η^2^_p_ = 0.03) was observed. More specifically, mean power over the 3 × WAnTs was higher during the Pref music condition compared to Non-pref (*p* = 0.044; d = 0.91). Furthermore, mean power during WAnT3 was significantly lower than WAnT1 (*p* = 0.016; d = 0.23). For total work (Joules; Figure 1b), there was a main effect for condition (*p* = 0.045; η^2^_p_ = 0.27) and test (*p* = 0.017; η^2^_p_ = 0.273). There was no interaction between condition × test (*p* = 0.634; η^2^_p_ = 0.03). In particular, total work over the 3 × WAnTs was higher during the Pref music condition compared to Non-pref (*p* = 0.045; d = 0.78). Total work during WAnT3 was significantly lower than WAnT1 (*p* = 0.016; d = 0.28).

### 3.2. Rate of Perceived Exertion (RPE) and Motivation

**Figure 2:**
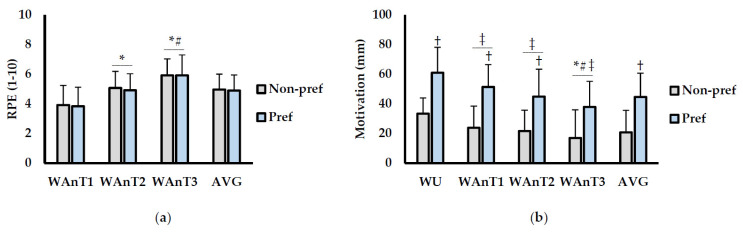
(**a**) Rate of perceived exertion (RPE; 1–10 scale) and (**b**) motivation (mm) between non-preferred (Non-Pref; grey bars) and preferred (Pref; blue bars) warm-up music conditions. Measurements are shown for WAnT1, WAnT2, WAnT3, and the average of all three tests together (AVG) for each condition. Data are presented as mean ± SD. * indicates significantly different from WAnT1 (*p* ≤ 0.05). # indicates significantly different from WAnT2 (*p* ≤ 0.05). ‡ indicates significant different from warm-up (WU) (*p* ≤ 0.05). † indicates significantly different than Non-pref (*p* ≤ 0.05).

RPE and motivation are displayed in (Figure 2). For RPE (1–10 scale; Figure 2a), there was a main effect for test (*p* < 0.001; η^2^_p_ = 0.74) but not for condition (*p* = 0.536; η^2^_p_ = 0.03). No interaction between condition × test (*p* = 0.0883; η^2^_p_ = 0.01) was observed. RPE was significantly higher during WAnT2 (*p* < 0.001; d = 0.89) and WAnT3 (*p* < 0.001; d = 1.55) compared to WAnT1. Furthermore, RPE was higher during WAnT3 compared to WAnT2 (*p* = 0.044; d = 0.80). For motivation (mm; Figure 2b), there was a main effect for condition (*p* < 0.001; η^2^_p_ = 0.64) and time (*p* < 0.001; η^2^_p_ = 0.58). There was also an interaction between condition × test (*p* = 0.048; η^2^_p_ = 0.14). Overall motivation was higher during the Pref music condition compared to Non-pref (*p* < 0.001; d = 1.55). More specifically, motivation was significantly higher during the Pref condition compared to the Non-pref following the WU (*p* = 0.034; d = 1.98), WAnT1 (*p* = 0.022; d = 1.85), and WAnT2 (*p* = 0.041; d = 1.42). Motivation was also significantly higher following the WU compared to WAnT1 (*p* = 0.022; d = 0.76), WAnT2 (*p* = 0.021; d = 1.30), and WAnT3 (*p* = 0.002; d = 1.56). Motivation during WAnT3 was also lower than in WAnT1 (*p* = 0.007; d = 0.80) and WAnT2 (*p* = 0.002; d = 0.42).

## 4. Discussion

Music preference has been shown to serve a pivotal role in the potency of ergogenic benefits of music during exercise, including warm-up music [1]. Pref warm-up music has been previously shown to improve both endurance rowing and resistance exercise performance [8,13]. However, it is unclear how warm-up music preference influences anaerobic exercise capacity. Thus, the current study aimed to explicate the effects of listening to Pref and Non-pref warm-up music on anaerobic sprint performance using repeated WAnTs. These findings reveal that listening to Pref warm-up music increases power output and total work versus Non-pref during repeated WAnTs. While RPE remained unchanged between conditions, motivation to exercise was higher throughout exercise following the listening of Pref warm-up music. Collectively, these results have important implications for preferred warm-up music selection to optimize performance, especially for collegiate athletes.

Current findings of increased performance with Pref warm-up music reinforce previous data from our lab [8,13]. Bolstering current improvements in mean power and total work over repeated sprints, we previously reported increased repetition volume over repeated sets of bench press exercises [8]. This may be, in part, due to increases in the anticipatory response to exercise. Indeed, warm-up music has been shown to increase both affective and autonomic responses prior to intense exercise. Chotorou et al. showed concomitant increases in vigor and sprint performance in trained individuals following a warm-up with music [4]. Furthermore, Yamamoto et al. reported increases in catecholamine anticipatory response with pre-exercise music, which could lead to enhanced muscle contractile function [20]. While not fully confirmed by current data, Pref music may lead to a heightened anticipatory response to exercise, allowing for greater effort and increased muscular force production compared to Non-pref music. In contrast, our group has also shown that listening to Pref music during repeated WAnTs did not result in improved power output [11]. This may be, in part, due to the timing of the music application. Due to the rhythmic nature of music, listening to music during exercise often results in a pacing effect. During rhythmic endurance-type exercise, synchronization of movement with music has been shown to increase exercise performance and efficiency [21,22]. However, due to the maximal nature of WAnTs, pacing to music may not only be non-beneficial but could be detrimental and undermine the benefits of Pref music. Since music was solely played during the warm-up in the current study, pacing ability may have been removed, thus allowing unencumbered maximal effort following listening to Pref warm-up music. Thus, disparities between findings may be due to the lack of a pacing effect suggesting Pref music may exhibit apical ergogenic effects during supramaximal exercise when played solely prior to the effort.

Observations of increased motivation to exercise with Pref music are well supported [1,10,11,12,13]. More specifically, Pref warm-up music has been shown to increase motivation during both endurance and resistance-based exercise [8,13]. Interestingly, regarding the current study, higher motivation following Pref warm-up music was sustained from immediately following the warm-up throughout the repeated WAnTs. This may have important practical implications for athletes and competitors. Since listening to music during competition is often unattainable, listening to music during a warm-up may allow for sustained motivation throughout repeated efforts during competition. Supporting this, McGuckian et al. recently showed that positive affective states were maintained throughout a soccer training session following listening to pre-exercise music [23]. However, how long the positive effects of Pref warm-up music persist is currently unknown and will necessitate future investigation to fully elucidate. Lack of differences in RPE between Pref and Non-pref warm-up music is also supported by previous investigations [8,13]. Listening to music during exercise has been well established to induce dissociation and lower RPE [24,25]. However, current findings further support the notion that the removal of musical stimuli during exercise eliminates dissociative effects. In totality, it appears that listening to Pref music prior to exercise, regardless of mode, does not result in favorable changes in RPE.

## 5. Limitations and Conclusions

While current findings present novel information regarding warm-up music preference and anaerobic performance, there were several limitations. First, only well-trained females were tested, thus not allowing for direct translations to other populations such as untrained, aged, or male counterparts. Given that previous evidence has suggested females may benefit from listening to music during repeated sprinting to a greater degree than males [26], more comprehensive and diverse samples are needed. Furthermore, not all intrinsic characteristics of the warm-up music (i.e., genre, lyrical content, etc.) were standardized. This area, in particular, is understudied, and further systematic investigations will be needed to dissect the potential roles of intrinsic factors of music.

In conclusion, Pref music improved power output and total work over repeated sprints in collegiate female athletes. Furthermore, RPE remained unchanged, but motivation was sustainably elevated during exercise following listening to Pref warm-up music. From a practical standpoint, athletes and competitors may benefit from ensuring they are listening to music they prefer during their warm-ups to optimize performance.

## Figures and Tables

**Table 1:** Descriptive characteristics (*n* = 14).

Characteristic	Mean ± SD
Age (yrs)	19.9 ± 1.3
Height (cm)	174.1 ± 10.7
Body mass (kg)	67.2 ± 11.1

## Data Availability

All data are available within this manuscript.
